# A Screening Method for the Isolation of Bacteria Capable of Degrading Toxic Steroidal Glycoalkaloids Present in Potato

**DOI:** 10.3389/fmicb.2018.02648

**Published:** 2018-11-05

**Authors:** Rosanna C. Hennessy, Niels O. G. Jørgensen, Carsten Scavenius, Jan. J. Enghild, Mathias Greve-Poulsen, Ole Bandsholm Sørensen, Peter Stougaard

**Affiliations:** ^1^Department of Plant and Environmental Sciences, University of Copenhagen, Frederiksberg, Denmark; ^2^Department of Molecular Biology and Genetics – Protein Science, Aarhus University, Aarhus, Denmark; ^3^KMC, Kartoffelmelcentralen, Amba, Brande, Denmark

**Keywords:** glycoalkaloids, α-chaconine, α-solanine, potato fruit juice, soil bacteria, *Arthrobacter*, microbial enzymes

## Abstract

Potato juice, a by-product of starch processing, is a potential high-value food ingredient due to its high protein content. However, conversion from feed to human protein requires the removal of the toxic antinutritional glycoalkaloids (GAs) α-chaconine and α-solanine. Detoxification by enzymatic removal could potentially provide an effective and environmentally friendly process for potato-derived food protein production. While degradation of GAs by microorganisms has been documented, there exists limited knowledge on the enzymes involved and in particular how bacteria degrade and metabolize GAs. Here we describe a series of methods for the isolation, screening, and selection of GA-degrading bacteria. Bacterial cultures from soils surrounding greened potatoes, including the potato peels, were established and select bacterial isolates were studied. Screening of bacterial crude extracts for the ability to hydrolyze GAs was performed using a combination of thin layer chromatography (TLC), high performance liquid chromatography (HPLC), and liquid chromatography mass spectrometry (LC-MS). Analysis of the 16S rRNA sequences revealed that bacteria within the genus *Arthrobacter* were among the most efficient GA-degrading strains.

## Introduction

Globally, plant-produced starch is a dominant nutrient source for millions of people, and among the most important starch-producing plants are potatoes (Ellis et al., 1999; Burrell, 2003; Jobling, 2004; Sonnewald and Kossmann, 2012). In addition to serving as a major nutrient source, potato starch is also an important industrial food ingredient. A by-product of the potato starch industry is potato fruit juice (PFJ) which contains proteins, amino acids and peptides (Zhang et al., 2017). While PFJ has a high nutritional value, vegetable proteins, including potato, contain many anti-nutritional components and thus must be refined prior to human consumption (Steinhof et al., 2005). In the case of potato proteins, the value is reduced due to the presence of toxic steroidal glycoalkaloids (GAs), polyphenols and the enzyme polyphenol oxidase (PPO) responsible for discolouration causing the final products to have a brown hue and consequently a negative affect on both solubility and digestibility (Prigent et al., 2007). GAs form part of the potato plants’ natural defense and have been shown to have toxic effects in humans causing among others gastrointestinal and neurological disorders (e.g., vomiting, diarrhea, headache, and hallucinations) (Grunenfelder et al., 2006). In addition, GAs add an undesirable bitter taste to potato protein-based products (Sinden et al., 1976). Consequently, these components must be removed from the potato protein fraction before it can be suitable for human consumption. Enzymatic removal of the carbohydrate moiety could provide a more sustainable and environmentally friendly process for detoxifying PFJ. A recent study by Koffi et al. (2017) demonstrated that a chemoenzymatic treatment of GAs using partial acidic hydrolysis combined with an enzymatic treatment enabled the efficient conversion of α-chaconine to solanidine. Solanidine is also a valuable product which can serve as a precursor for hormone synthesis and other pharmacologically active compounds (Vronen et al., 2003; Koffi et al., 2017). The enzyme used in the study was a β-glycosidase from the cockroach *Periplaneta americana* (Koffi et al., 2017). To that end, we wanted to identify bacterial isolates capable of producing specific enzymes capable of degrading toxic potato-derived GAs into solanidine.

It has previously been established that both potato-derived extracts and extracts of fungal derived pathogens contain GA-degrading activities (Weltring et al., 1997; Oda et al., 2002; Nikolic et al., 2005; Dahlin et al., 2017). Some of these enzymes appear to act by sequential degradation of the carbohydrate moiety, indicating that multiple enzymes may be required for complete deglycosylation of the alkaloid. Furthermore, the enzymes that can degrade alkaloids may be specific for only one of the two main GAs produced in potato, α-chaconine and α-solanine. These compounds are found in all parts of the plant and are typically produced by plants as biopesticides, or when the plant is damaged. Potato GAs consist of the solanidine aglycone with a carbohydrate side-chain, which is important for mediating interactions with cell membranes (Koffi et al., 2017). It has been proposed that in order to detoxify α-chaconine and α-solanine, the first step is removal of the trisaccharide to release solanidine. However, a recent study showed solanidine to have a great impact on the physiology of the phytopathogen *Phytophthora infestans* compared to its glycosylated form (Dahlin et al., 2017). Only scarce information and few papers currently describe the microbes and enzymes involved in the degradation of potato GAs and therefore new knowledge is urgently needed. To begin to fill this information gap we here describe a series of methods for the identification of GA-degrading bacteria.

In this study, we aimed to develop a pipeline for the isolation, screening and selection of bacteria capable of degrading α-chaconine and α-solanine. Enrichment cultures were established to isolate bacterial strains capable of tolerating high concentrations of GAs or of utilizing them as a sole carbon source. Using a combination of low-tech [enrichment cultures, thin layer chromatography (TLC)] and high-tech [liquid chromatography mass spectrometry (LC-MS)] techniques we successfully isolated and screened bacterial strains capable of degrading GAs demonstrating the utility of this pipeline as a starting point for enzyme discovery. To the best of our knowledge, this is the first report describing bacteria capable of degrading potato GAs.

## Materials and Methods

### Extraction of α-Chaconine and α-Solanine and Preparation of Standards

Crude extraction of (GAs) from potato protein supplied by Kartoffelmelcentralen (KMC), Amba was performed as follows: 0.5 g of potato protein was added to 5 ml methanol-water-acetic acid (at a ratio of 80:9.5:0.5) in a 50 ml tube and shaken vigorously for 10 min. The tube was centrifuged 10 min at 4°C and the upper phase gently decanted into a fresh 50 ml tube. Extractions were pooled and dried in a vacuum centrifuge before resuspension in 1.5 ml filter-sterilized potassium phosphate buffer at pH 7.5. To improve the solubility of the GAs, 1.5 μl acetic acid was added. The crude extract was filter-sterilized using a 0.45 μm filter after which the purified GAs were detected and identified by HPLC analysis (see below).

Pure α-chaconine was obtained from PhytoLabs (Vestenbergsgreuth, Germany) and α-solanine was purchased from either PhytoLabs or Sigma-Aldrich (Brøndby, Denmark).

### Isolation and Purification of Bacterial Strains From Potato Soil Extracts

Bacteria were isolated on potato dextrose agar at fifth strength (PDA 1/5) by inoculation of material from greened potatoes and the surrounding soil. Alternatively, minimal media as described by Nan and Edens, 1998 was supplemented with a pure or crude extraction of GAs (Supplementary Figure S1) was used for isolation of bacteria. Plates were incubated at 15°C for a week and bacterial colonies were isolated for purification. Following three rounds of isolation to obtain pure cultures, selected strains were routinely cultured in either Lysogeny broth (LB) or on LB agar at 20°C for 1–2 days, depending on the strain.

### Screening for Bacteria Capable of Using α-Chaconine or α-Solanine as a Sole Carbon Source

To determine whether the bacterial isolates could use the GAs as a sole carbon source, the strains were grown overnight in LB at 20°C and 5 μl culture plated on Petri dishes containing minimal media as described above enriched with either α-chaconine or α-solanine as a sole carbon source. Strains that scored positive for growth were selected for further characterization.

### DNA Extraction and Determination of 16S Ribosomal RNA Sequences

Extraction of crude DNA from the isolated bacterial cultures was performed as follows. Bacterial cells were scraped off a Petri dish and resuspended in 1 ml sterile phosphate-buffered saline (1 × PBS) buffer in a sterile Eppendorf tube. The cells were concentrated by centrifugation for 5 min at 10,000 × *g* after which the supernatant removed. For extraction of DNA, the pellet was resuspended in 100 μl sterile water and boiled 10 min and immediately placed on ice before adding 900 μl ice-cold sterile water. Cell debris was pelleted by centrifugation for 5 min at 10,000 × *g*. The supernatant containing DNA was transferred to a clean tube ready for PCR analysis or storage at −20°C. The 16S rRNA gene sequence was determined by PCR using the universal bacterial 16S rRNA primers: 16S-27F (5′- AGA GTT TGA TCM TGG CTC AG-3′) and 16S-1492R (5′- CGG TTA CCT TGT TAC GAC TT-3′) (Webster et al., 2006). Sanger sequencing of the 16S rRNA gene was performed by GATC-Biotech, Konstanz, Germany, for the identification of select strains.

### Enzyme Assays

For preliminary screening, strains were grown overnight in 5 ml LB shaking at 200 rpm at 20°C and then diluted 1:100 into LB medium either water (control) or 10 μg ml^−1^ final concentration of α-chaconine or α-solanine and incubated 20 h under the same conditions. To obtain crude extracellular extracts, cultures were centrifuged at 20°C for 30 min at 4000 × *g* and either used directly or frozen at −20°C. Enzyme reactions consisting of 10 μl 50 mM sodium acetate buffer pH 5.5, 40 μl substrate (0.2 mM GA in 100 mM sodium acetate buffer pH 5.0) and 45 μl of crude extract was set up and incubated 24 h at 20°C. Enzyme reactions were terminated by heating for 10 min at 90°C. After centrifugation or 10 min at 17,000 × *g*, the supernatant was transferred to a fresh 1.5 ml tube and dried in a vacuum for further analysis. Pellets were resuspended in 20 μl of methanol and 8 μl were spotted on TLC plates for analysis or 10 μl were characterized by HPLC analysis.

For selecting strains for further characterization, strains were grown overnight in 5 ml LB shaking 200 rpm 20°C and then washed with 0.9% NaCl and standardized to an OD_600__nm_ of 1.0. To 100 ml shake flask, 1 ml of bacterial culture (final OD_600nm_ of 0.1) was added to 9 ml LB supplemented with acidic phosphate buffer (control) or a glycoalkaloid mix (5 μg ml^−1^ each of α-chaconine, α-solanine, and α-tomatine) and grown shaking 200 rpm 20°C for 20 h. To obtain crude extracellular extracts, cultures were centrifuged at 20°C for 20 min at 4000 × *g* and used directly for enzyme activity analysis. Enzyme assays consisting of 10 μl 50 mM sodium acetate buffer pH 5.5, 40 μl substrate (0.2 mM of α-chaconine and 0.2 mM of α-solanine) in 100 mM sodium acetate buffer pH 5.0 and 45 μl of crude extract were set up and incubated at 20°C for 35 h. Enzyme reactions were terminated by heating for 10 min at 90°C and subsequently centrifuged for 10 min at 17,000 × *g* before transferring the supernatant to either an HPLC vial (60 μl) for semi-quantitative analysis or to a fresh 1.5 ml tube (30 μl) for qualitative TLC analysis. For TLC analysis, 30 μl extract was dried 1 h in a vacuum centrifuge and resuspended in 7 μl cold methanol. Samples were briefly centrifuged and 5 μl spotted on TLC plate. TLC plates were run twice in *n*-butanol-acetic acid-water (2:1:1) solvent system and sprayed with 50% vv^−1^ H_2_SO_4_ and heated for 10 min at 85°C. For HPLC analysis, a 25 μl sample was injected and conditions for analysis were as described below. For analysis of intermediate metabolites produced during degradation or detection of solanidine, 30 μl of sample was analyzed by LC-MS.

### Thin Layer Chromatography (TLC)

TLC was performed on silica gels 60 F254 (Merck) using *n*-butanol-acetic acid-water (2:1:1) as a running solvent and plates were developed on a hot plate after spraying with 50% vv^−1^ H_2_SO_4_.

### High Performance Liquid Chromatography (HPLC) Analysis

Quantification and identification of α-chaconine and α-solanine were performed using a Waters 2695 Alliance system with Waters 2487 Dual absorbance detector at 202 nm. The column was a Waters Nova-Pak C_18_ with 4 μm particle size, operated at 40°C. Solvent were 60% acetonitrile and 40% phosphate buffer at pH 7.6 (mixture of K_2_HPO_4_ and KH_2_PO_4_ to obtain the correct pH value) at a flow rate of 1.5 ml/min.

### Liquid Chromatography Mass Spectrometry (LC-MS) Analysis

Quantification and identification of α-chaconine and α-solanine was based on the ion intensity of the single charged monoisotopic mass (α-chaconine, m/z 852.51; α-solanine, m/z 868.51). The presence of solanidine was monitored at m/z 398.34. Ion intensities were normalized to the level in untreated LB media. Samples were analyzed in an LC-MS setup with an eksigent nanoLC 415 system (Sciex) connected to a TripleTOF 6600 mass spectrometer (Sciex) equipped with a NanoSpray III source (AB Sciex). Ion-intensities were extracted in Pekaview 1.2 (Sciex).

## Results and Discussion

**FIGURE 1:**
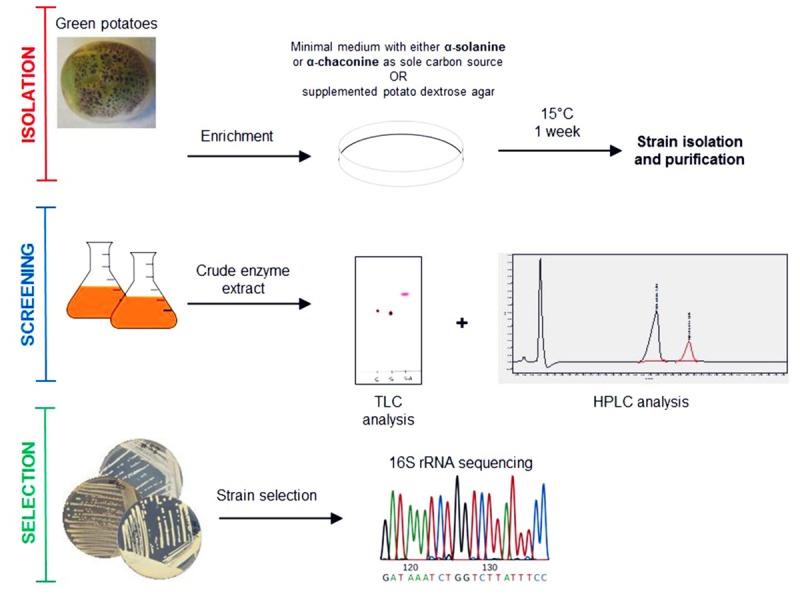
Isolation scheme for glycoalkaloid (GA) degrading bacteria.

**FIGURE 2:**
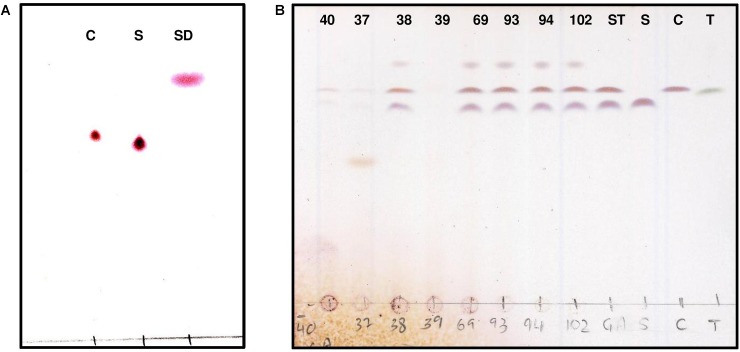
Thin layer chromatography (TLC) detection of purified GA from potato α-chaconine and α-solanine. TLC analysis of hydrolysis products was used to visualize the standards **(A)** α-chaconine (C), α-solanine (S) and solanidine (SD). Developed TLC method was used to visualize the degradation products following GA hydrolysis by bacterial crude extracts (strains 40, 37, 38, 39, 69, 93, 94, and 102) obtained from pure cultures **(B)** compared to α-chaconine (C), α-solanine (S) and α-tomatine (T).

**FIGURE 3:**
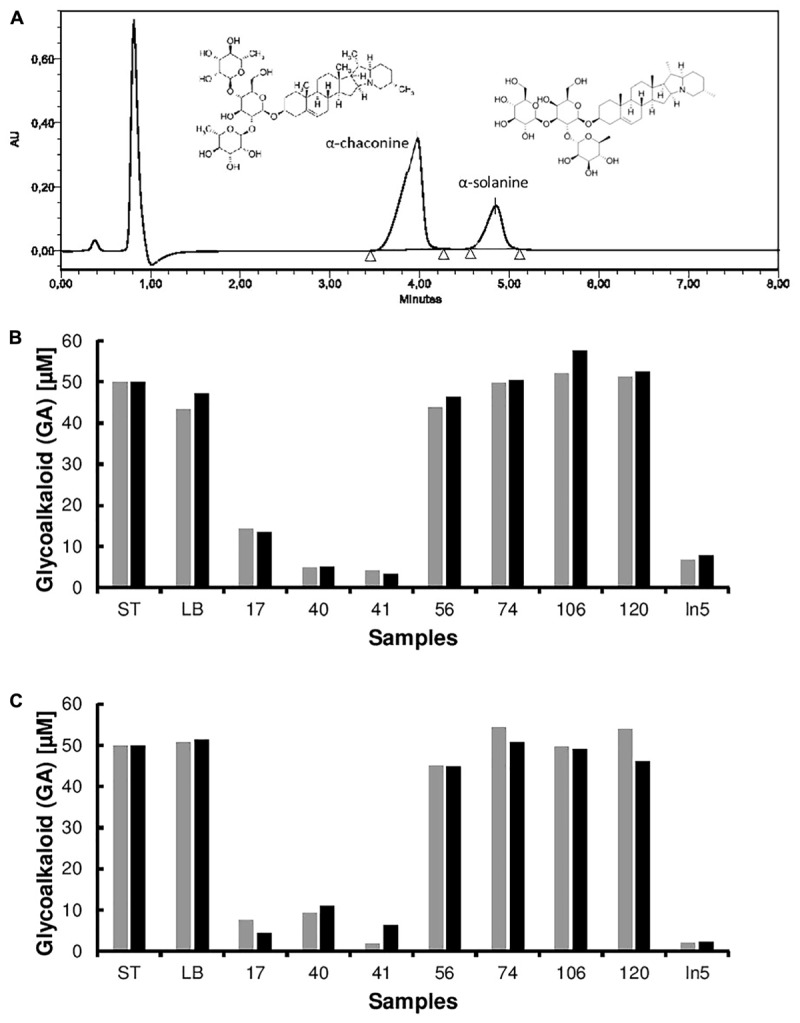
High performance liquid chromatography (HPLC) spectra of the pure glycoalkaloids (GA) α-chaconine and α-solanine from potato. HPLC analysis of hydrolysis products was used to quantify GA degradation by bacterial extracts from pure cultures **(A)**. Degradation of potato-derived glycoalkaloids α-chaconine (black) and α-solanine (gray) by crude extracts obtained from bacterial cell cultures (strains 17, 40, 41, 56, 74, 106, 120, and In5) compared to the control (media blank LB) and standard (ST) induced **(B)** or non-induced **(C)**.

A previous study demonstrated a rapid turnover of potato (GAs) in top soils of Danish potato fields, presumably due to microbial metabolism (Jensen et al., 2009). Therefore, it is likely that bacteria producing GA-degrading enzymes are present in such environments. In addition, green potato peel is known to contain significantly higher amounts of toxic GAs (Omayio et al., 2016). Based on these observations, a screening strategy for GA-degrading bacteria was established as shown in Figure 1. Potato extracts from green potato peel and the immediate surrounding soil were used as a potential source of GA-degrading bacteria. To select for such bacteria, a series of enrichment cultures were established using commercially purified GAs or a crude extract produced from potato powder (Supplementary Figure S1). A crude preparation of GAs was made as pure α-chaconine and α-solanine are expensive products. Hence, when using pure compounds small petri dishes with a small volume of media were used. From these enrichment cultures a total of 85 isolates were obtained and used for further analysis. To determine whether the isolated strains could utilize GAs as a sole carbon source, cultures grown overnight were transferred to petri dishes with minimal medium and supplemented with either α-chaconine or α-solanine as a sole carbon source. A total of 14 strains that grew on the GA-enriched media were transferred to liquid media containing either a mixture of GAs or no GAs and cultured overnight. Crude bacterial extracts were screened for GA degradation using an optimized and rapid TLC method for the detection of both α-chaconine or α-solanine (Figure 2 and Supplementary Figure S2). In addition to these two compounds, the aglycone solanidine that is produced upon removal of the carbohydrate moiety of the two compounds, was included in the TLC analysis (Figure 2). Parallel to the TLC analysis, GA degradation was also confirmed by HPLC and LC-MS analysis, respectively (Figure 3 and Supplementary Figures S3, S4). Interestingly, no solanidine was detected in samples where bacterial extracts were incubated with GAs, indicating that the isolates may be capable of the complete hydrolysis and metabolism of GAs and their degradation products. The only GA related intermediate product detected by LC-MS was a 705.5 Da GA variant corresponding to either α-chaconine or α-solanine were the outermost saccharide have been removed by hydrolysis resulting in solanidine modified with a disaccharide (Supplementary Figure S4).

Of the 14 GA degrading bacterial strains isolated from a series of enrichment cultures, four strains S37, S39, S40, and S41, were the best at degrading potato GAs. Unsurprisingly, the four strains were all isolated from minimal media solidified with agar containing a mixture of pure α-chaconine and α-solanine as a sole carbon source. Enrichment cultures containing GAs as a sole carbon source was the best method to enable the isolation of GA degrading bacteria.

While the microbial degradation of GAs is known, few studies report bacteria capable of degrading GAs. For example, *Clavibacter michiganensis* and *Streptomyces scabies* are bacteria that can degrade the tomato GA α-tomatine, however, to the best of our knowledge no studies report the bacterial degradation of potato GAs (Kaup et al., 2005; Seipke and Loria, 2008). During screening of the strain collection, we observed that one strain, *Serratia* sp. S40 also displayed antifungal activity. In order to investigate whether other biocontrol bacteria can degrade GAs, we tested the ability of the well-described antifungal strain *Pseudomonas fluorescens* In5 isolated from a potato field in southern Greenland (Michelsen and Stougaard, 2011; Hennessy et al., 2017). We observed that *P. fluorescens* In5 was able to degrade both α-chaconine and α-solanine and similar to the GA-degraders described in this study, appeared to be capable of the complete degradation and metabolism of both compounds as no solanidine was detected following hydrolysis of the GAs by a crude enzyme extract from *P. fluorescens* In5. Further studies investigating the mechanisms by which these bacteria degrade GAs will be required.

Identity of the four strains that produced GA-degrading extracts was done by analysis of 16S rRNA gene sequences (Table 1). Three isolates (S37, S39, and S41) showed more than 99% sequence identity to species of the genus *Arthrobacter* and the fourth isolate (S40) showed 98% sequence identity to species of the genus *Serratia*. All strains were capable of degrading both α-chaconine and α-solanine. Published information on GA-degrading bacteria is sparse, but it has previously been reported that an α-chaconinase enzyme produced by *Gibberella pulicaris* is induced by α-chaconine, α-solanine and the tomato glycoalkaloid α-tomatine (Becker and Weltring, 1998). All four strains were able to degrade α-chaconine and α-solanine independent from whether bacterial cultures were induced by the addition of GAs. Further characterization of the four selected strains is now necessary to determine their species and elucidate the enzymes involved in GA degradation.

**Table 1:** 16S ribosomal RNA sequences of isolates.

Isolate	Closest relative	Genbank accession	Identity scores (%)
37	*Arthrobacter* sp. PJ8	MH245050	99
39	*Arthrobacter* sp. X49	MH245051	99
40	*Serratia* sp. Y4	MH245052	98
41	*Arthrobacter* sp. PJ8	MH245053	99

The isolation method here described identified four strains, which demonstrated the ability to metabolize and degrade the potato-derived GAs α-chaconine and α-solanine. The 16S rRNA gene sequences of these strains showed highest similarity to strains belonging to genera *Arthrobacter* and *Serratia*. Functional genomics of the identified strains is now required to identify the genes and metabolic pathways related to the biodegradation of potato GAs.

## Conclusion

We successfully developed a screening method to isolate GA-degrading bacteria from potato soil extracts. This will enable the future identification and characterization of enzymes involved in the degradation of GAs to upgrade industrial waste products into valuable food ingredients.

## Author Contributions

RH and PS conceived the study. RH, NJ, and CS performed the experiments. All authors analyzed and interpreted the data, drafted and approved the manuscript.

## Conflict of Interest Statement

The authors declare that the research was conducted in the absence of any commercial or financial relationships that could be construed as a potential conflict of interest.
