# Supplementary figures and images for: A Screening Method for the Isolation of Bacteria Capable of Degrading Toxic Steroidal Glycoalkaloids Present in Potato
Source: Front Microbiol. 2018 Nov 5;9:2648. doi: 10.3389/fmicb.2018.02648 (PMC6230958; doi:10.3389/fmicb.2018.02648)

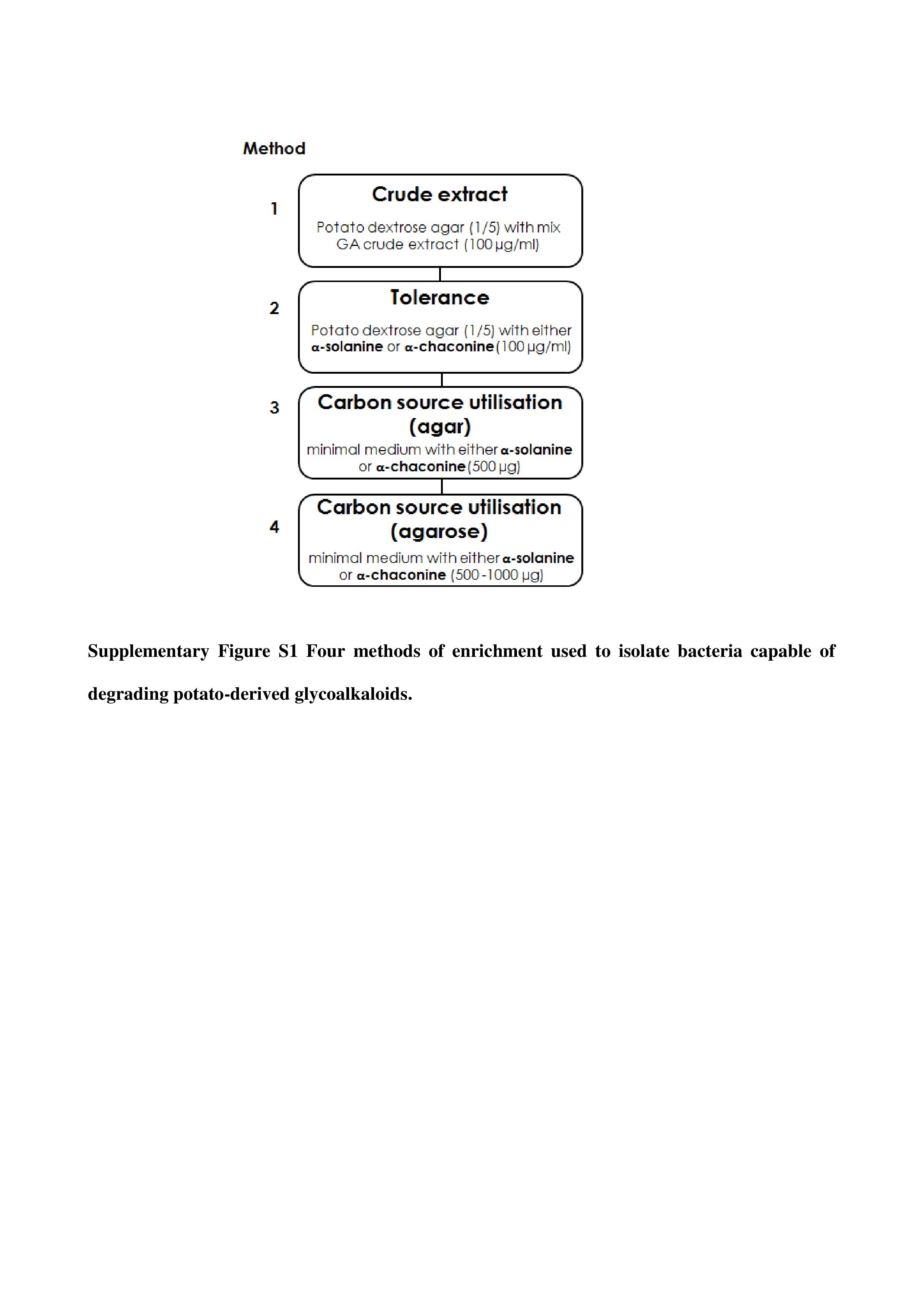

Supplement: Supplementary file 1 [file Image_1.JPEG]

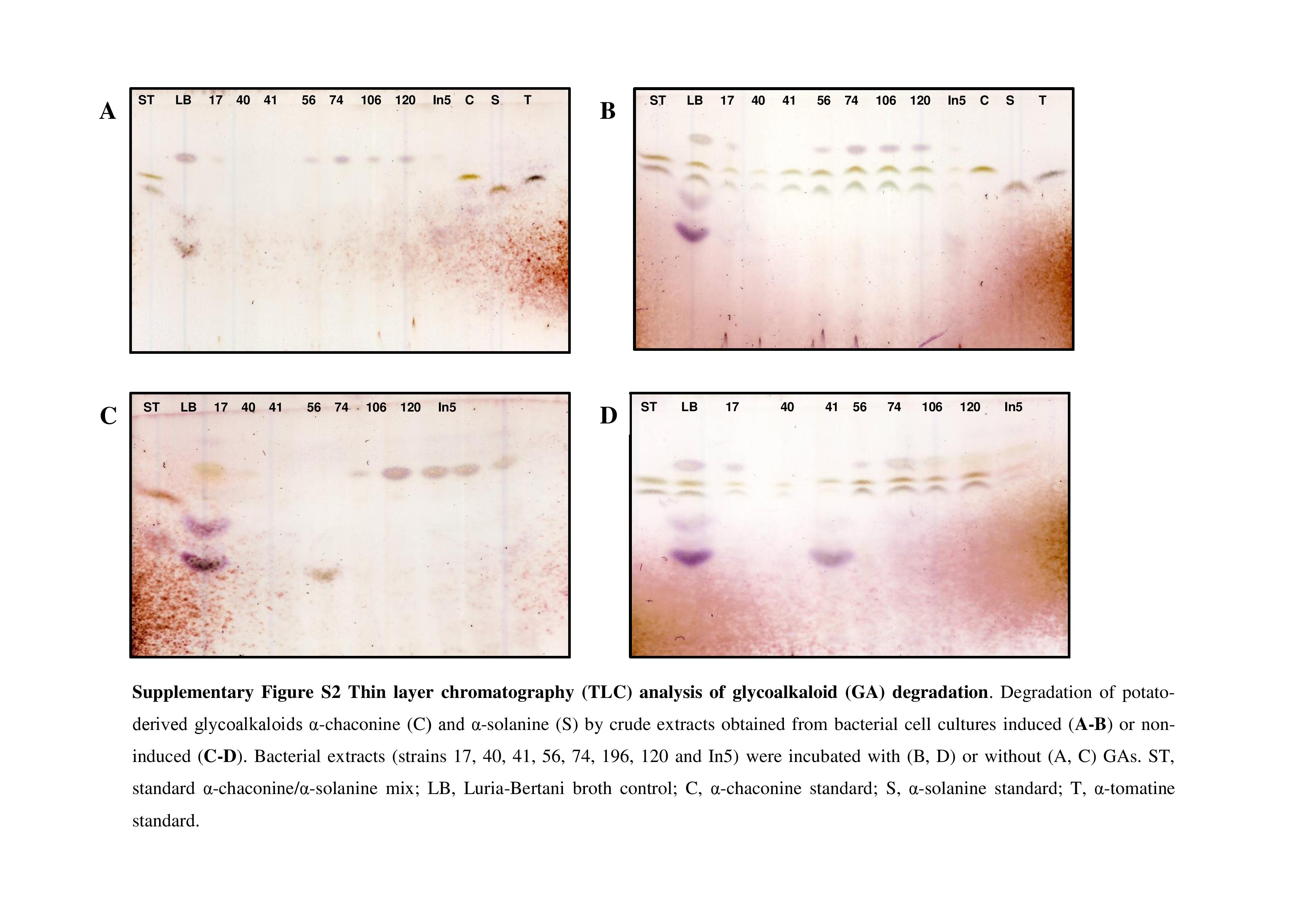

Supplement: Supplementary file 2 [file Image_2.JPEG]

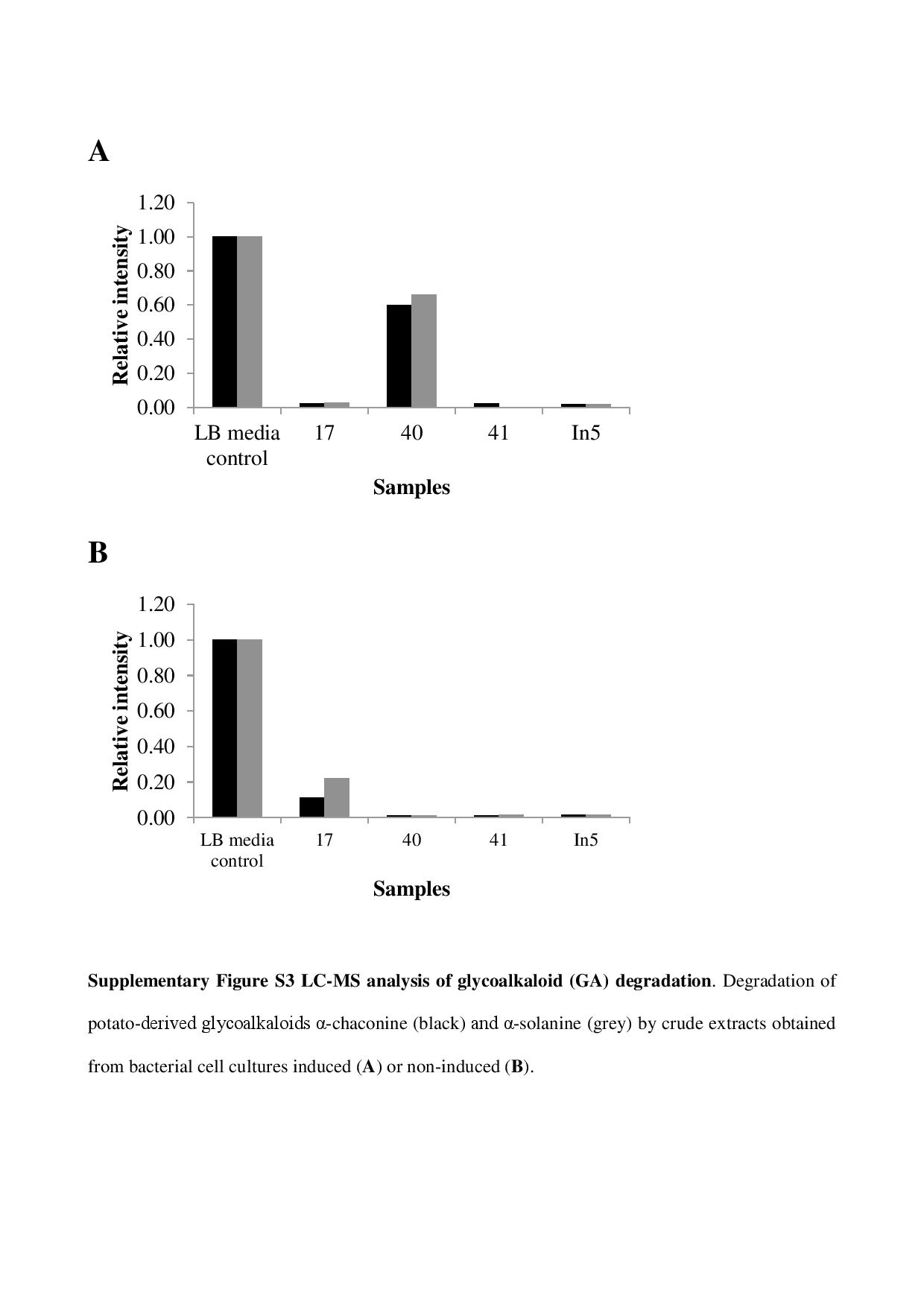

Supplement: Supplementary file 3 [file Image_3.JPEG]

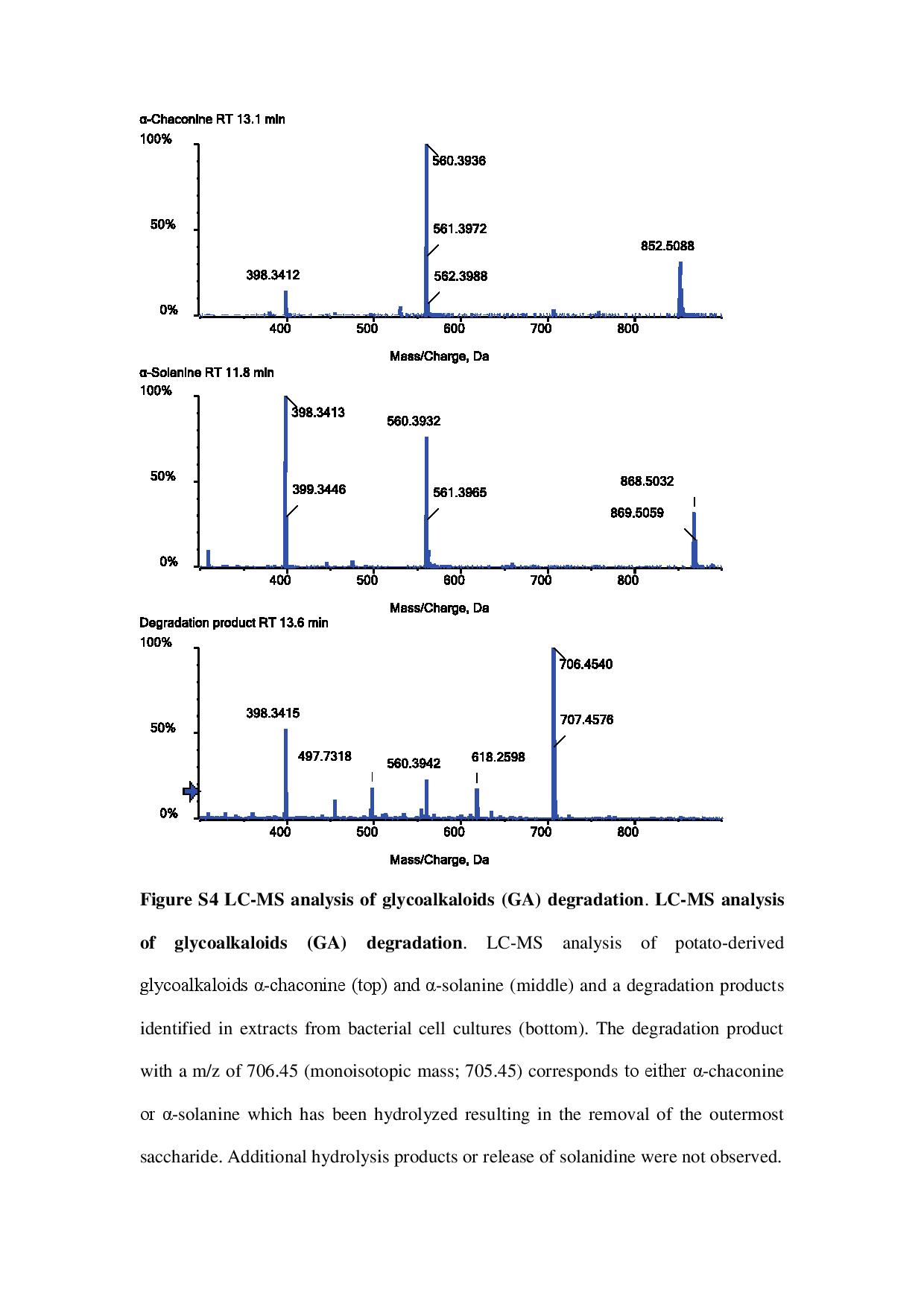

Supplement: Supplementary file 4 [file Image_4.JPEG]
